# Sunscreen for Fish: Co-Option of UV Light Protection for Camouflage

**DOI:** 10.1371/journal.pone.0087372

**Published:** 2014-01-29

**Authors:** Kaspar P. Mueller, Stephan C. F. Neuhauss

**Affiliations:** University of Zurich, Institute of Molecular Life Sciences, Neuroscience Center Zurich and Center for Integrative Human Physiology, Zurich, Switzerland; Purdue University, United States of America

## Abstract

Many animals change their body pigmentation according to illumination of their environment. In aquatic vertebrates, this reaction is mediated through aggregation or dispersion of melanin-filled vesicles (melanosomes) in dermal pigment cells (melanophores). The adaptive value of this behavior is usually seen in camouflage by allowing the animal to visually blend into the background. When exposed to visible light from below, however, dark-adapted zebrafish embryos at the age of 2 days post fertilization (dpf) surprisingly display dispersal instead of aggregation of melanosomes, i.e. their body coloration becomes dark on a bright background. Melanosomes of older embryos and early larvae (3–5 dpf) on the other hand aggregate as expected under these conditions. Here we provide an explanation to this puzzling finding: Melanosome dispersion in larvae 3 dpf and older is efficiently triggered by ultraviolet (UV) light, irrespective of the visual background, suggesting that the extent of pigmentation is a trade-off between threats from predation and UV irradiation. The UV light-induced dispersion of melanosomes thereby is dependent on input from retinal short wavelength-sensitive (SWS) cone photoreceptors. In young embryos still lacking a functional retina, protection from UV light predominates, and light triggers a dispersal of melanosomes via photoreceptors intrinsic to the melanophores, regardless of the actual UV content. In older embryos and early larvae with functional retinal photoreceptors in contrast, this light-induced dispersion is counteracted by a delayed aggregation in the absence of UV light. These data suggest that the primary function of melanosome dispersal has evolved as a protective adaption to prevent UV damage, which was only later co-opted for camouflage.

## Introduction

Many invertebrates and lower vertebrates including reptiles, amphibians and fish are able to adapt their body pigmentation to the color of the local environment [1], [2]. This behavior, termed visual background adaptation, is also observed in larval zebrafish (*Danio rerio;* [3]). In aquatic vertebrates in general, this reaction is mediated through aggregation or dispersion of melanosomes (melanin-filled vesicles) residing in dermal pigment cells called melanophores. The melanosomes thereby are transported along microtubule tracks and actin filaments by employing different molecular motors [4], [5]. The minus-end directed motor protein dynein is responsible for carrying the melanosomes towards the center of the cell where they aggregate, giving the animal a pale appearance [6], [7]. In order to adapt their body color to a dark background, melanosomes are dispersed by employment of the plus-end directed motor kinesin-2, which carries the melanosomes towards the periphery of the cell [8], and myosin V, which switches the melanosomes from the microtubule tracks to actin filaments, ensuring an even dispersion throughout the cell [5], [9].

For different animals including the zebrafish, it has been shown that visual background adaptation depends on retinal input, since blinded animals most often fail to adapt their body color to the background (refer to e.g. [10]–[16]). A lack of visual background adaptation therefore can give a first hint for a visual defect. Indeed, a number of mutant zebrafish strains initially identified by their dark appearance were shown to be blind in subsequent behavioral tests [17].

The adaptive value of visual background adaptation is commonly seen in camouflage by allowing the animal to blend with the hue of the local environment and thereby avoid visual detection by predators. However, this idea contravenes the finding that freshly hatched zebrafish embryos at the age of 2 days post fertilization (dpf) show dispersion of melanosomes when exposed to visible light from below, i.e. their body color becomes dark on a bright background [18]. In older larvae, however, the course of pigmentation change upon illumination with visible light markedly differs. Starting at 3 dpf, white light still induces an initial, rapid dispersion of melanosomes, but in contrast is followed by a slower aggregation and consequently leads to the expected paling of body color [18]. The unexpected light-induced dispersion of melanosomes in young zebrafish embryos raises the question whether body pigmentation in zebrafish may serve an additional function, namely to protect the transparent larvae from the impact of ultraviolet (UV) irradiation.

In this work, we tested this hypothesis by comparing pigmentation change of wildtype and mutant zebrafish larvae at different developmental stages in response to illumination with visible and UV light. Our data suggest that indeed the primary function of pigments in young zebrafish embryos is not to provide camouflage, but rather to protect these thin-skinned animals from UV irradiation. Only in older larvae and in the absence of UV light, melanosome dispersion and aggregation serves a camouflage function, while under the influence of UV light, protection from UV-induced damages remains predominant.

## Materials and Methods

Animal care and all experimental procedures were carried out in accordance with the European Communities Council Directive (86/609/EEC). All experiments were approved and performed according to animal care regulation of the Kantonale Veterinäramt Zürich.

### Fish Maintenance and Breeding

Fish were maintained and bred as previously described [19]. Larvae were kept on a 14/10 h light/dark cycle at 28°C in E3 medium (5 mM NaCl, 0.17 mM KCl, 0.33 mM CaCl_2_ and 0.33 mM MgSO_4_). All experiments were carried out between 9 A.M. and 8 P.M.

### Measurement of Visible- and UV-light Induced Change of Body Pigmentation

Larvae between 2 and 5 dpf were transferred to a 35 mm Petri dish and excess E3 medium was removed. Larvae were then embedded in ∼2 ml of 2% low melting temperature agarose (NuSieve GTG agarose; Cambrex BioScience, Rockland, ME) and aligned to lay dorsal side up. After bonding, the agarose was laid over with ∼2 ml E3 medium. The 35 mm Petri dish subsequently was bathed in a 9 cm dish filled with E3 medium and placed under a dissecting microscope (SV8; Zeiss, Oberkochen, Germany).

**Figure 1:**
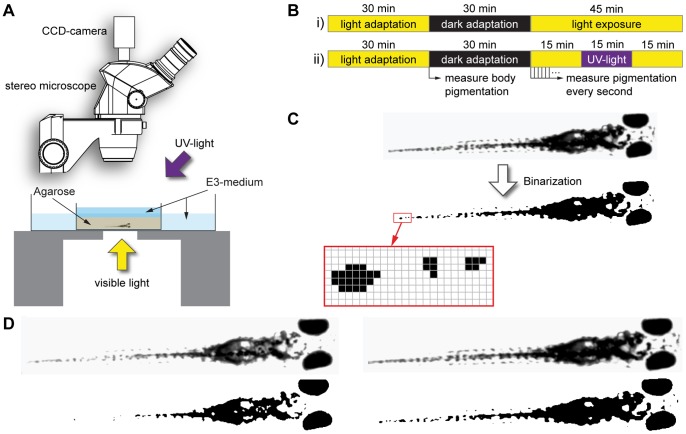
Experimental procedure. A) Schematic drawing of the experimental setup. Zebrafish larvae are embedded in agarose and placed under a dissecting microscope. Illumination with visible light is provided from below, optional near-ultraviolet light from obliquely above. B) After an initial light adaptation period lasting 30 minutes, the extent of body pigmentation is measured and serves as a reference for all following measurements. The larvae are then dark adapted for another 30 minutes, after which they are exposed to visible light for 45 minutes (i). During light exposure, the extent of pigmentation is measured every second. Optional UV light is provided during a 15 minutes interval in the middle of the light exposure phase (ii). C) To quantify the extent of body pigmentation, images of the larva are binarized using a fixed threshold. The number of pixels above this threshold is summed and divided by the number of pixels above threshold after 30 minutes light adaptation, resulting in a “melanin index”. D) Example original (top row) and binarized (bottom row) images of the same 5 day old zebrafish larva with minimal melanosome dispersion after dark adaptation (left, melanin index = 1.14) and maximal melanosome dispersion reached after 15 minutes of UV exposure (right, melanin index = 1.98).

Visible light was provided from below (∼13 klx; KL 1500; Schott, Mainz, Germany). Additional near-UV light was provided from obliquely above by a UV-spot (No. 51100700; Eurolite, Waldbuettelbrunn, Germany; equipped with an Omnilux UV energy saving lamp, 25 W) mounted at a distance of 15 cm in front of the larva, resulting in an irradiance of ∼1.5 W/cm^2^ at the position of the larva (Fig. 1A).

In a typical experiment, larvae were illuminated with white light for 30 min for light adaptation. The light was then switched off and the larvae were left in complete darkness for another 30 min for dark adaptation. Afterwards, white light was presented again for 45 min in total (Fig. 1Bi).

In all experiments using UV-light, the UV lamp was switched on after the first 15 min of exposure with visible light and presented for 15 min. For the last 15 min, the larvae were again illuminated with visible light only (Fig. 1Bii).

Images of the larva were taken at 1 frame per second using a CCD-camera (XC-75 CE; Sony Corporation, Tokyo, Japan) attached to the dissecting microscope. The images were binarized using custom-made software based on NI LabView 2009 and NI Vision Development Module 2009 (National Instruments, Austin, TX). All pixels above a fixed threshold were summed to yield a measure of the total area of dark body parts (Fig. 1C).

For plotting the data of the light induced body color change, measurements of each larva were normalized to the values obtained after 30 min light adaptation. Plots were generated using R 2.9.2 (www.r-project.org).

### Eye Excision and Tail Isolation

4 dpf larvae were briefly anesthetized in 200 mg/l MS-222 (Sigma-Aldrich, St. Louis, MO) and transferred to a piece of tissue. Both eyes were excised under a dissecting microscope using stainless needles with a diameter of 0.1 mm. The larvae were then transferred into Ringer’s solution (111 mM NaCl, 2.5 mM KCl, 1.6 mM MgCl_2_, 1.0 mM CaCl_2_, 0.01 mM EDTA, 3 mM HEPES; adjusted to pH 7.72) and left to recover for ∼24 h.

Tails of anesthetized larvae were cut off posterior of the yolk sacs or swim bladders, respectively, using a razor blade. Isolated tails were transferred into Ringer’s solution and left to recover for ∼2 h. Embedding and measurement of pigmentation change of enucleated larvae and isolated tails was done in the same way as for intact larvae.

## Results

### Melanosome Dispersion in Larval Zebrafish is Efficiently Triggered by UV Light

**Figure 2:**
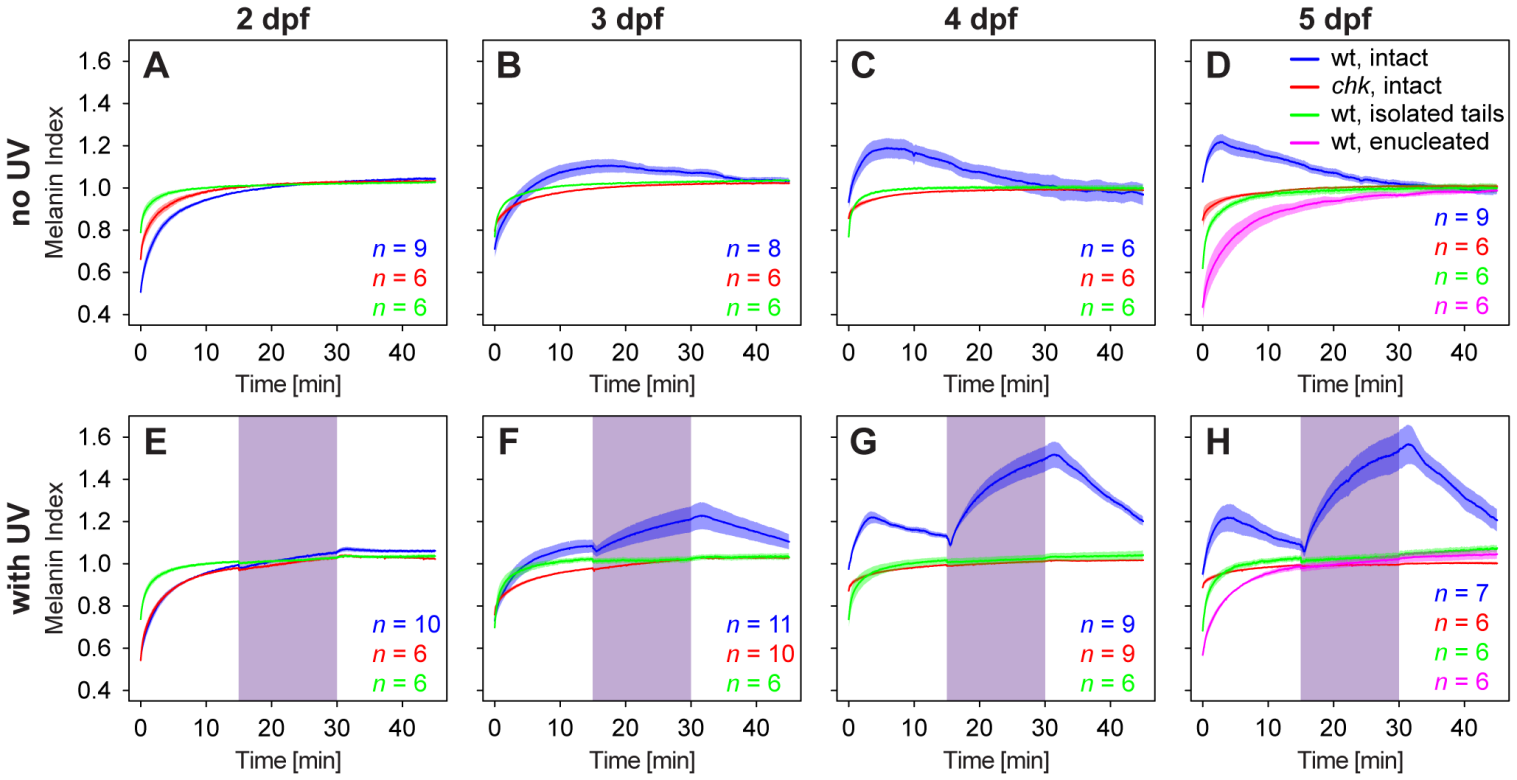
Influence of ocular photoreceptors on pigmentation of larval zebrafish. Similar to the situation found in wildtype embryos at 2*chk* mutant larvae, of blinded wildtype larvae as well as of isolated tails disperse upon stimulation with visible light (A–D) and show no additional reaction to UV light (E–H). In contrast, visible light induces a delayed aggregation of melanosomes in wildtype intact larvae of 3 dpf and older (B–D) while exposure of these larvae to UV light leads to a pronounced dispersion of melanosomes (F–H). Values represent means ±1 SEM and are relative to the extent of pigmentation after 30 minutes light adaptation, i.e. a melanin index >1 means the larva is darker compared to the appearance after light adaptation, and vice versa, a value <1 means the larva appears paler compared to the appearance after light adaptation. For ease of comparison, all graphs are drawn at the same scale. Purple shaded areas mark the time interval when the UV light was on.

As previously reported [18], the melanosomes in the melanophores of wildtype zebrafish embryos at the age of 2 days post fertilization (dpf) aggregate during dark adaptation, leading to a pale appearance of the embryos. Upon subsequent exposure to visible light from below, the melanosomes rapidly disperse, leading to a pronounced darkening of body color on a bright background (Fig. 2A, blue line). Similar results were obtained from chokh *(chk)* mutant embryos, completely lacking their eyes due to a mutation in the homeodomain-containing transcription factor Rx3 ([20]; Fig. 2A, red line). Hence the observed reaction is not dependent on retinal input, and even takes place in isolated tails from wildtype embryos (Fig. 2A, green line). Addition of UV light during exposure with visible light has no additional effect at this embryonic stage (Fig. 2E, blue line). Starting from 3 dpf however, the initial period of fast melanosome dispersal upon exposure to visible light is followed by a delayed, slower aggregation of melanosomes in wildtype embryos and early larvae (Fig. 2B–D, blue line). This delayed aggregation is neither present in *chk* mutants, nor in isolated tails nor wildtype larvae with surgically removed eyes (Fig. 2B–D, red, green and magenta lines, respectively). Addition of UV light from above leads to a fast and pronounced dispersal of melanosomes in sighted embryos and larvae from 3 dpf onwards (Fig. 2F–H, blue line), but has neither an effect in blind larvae nor in isolated tails (Fig. 2F–H, red, green and magenta lines, respectively).

### UV Light Induced Dispersal of Melanosomes is Mediated by Short Wavelength-sensitive (SWS) Cone Photoreceptors

**Figure 3:**
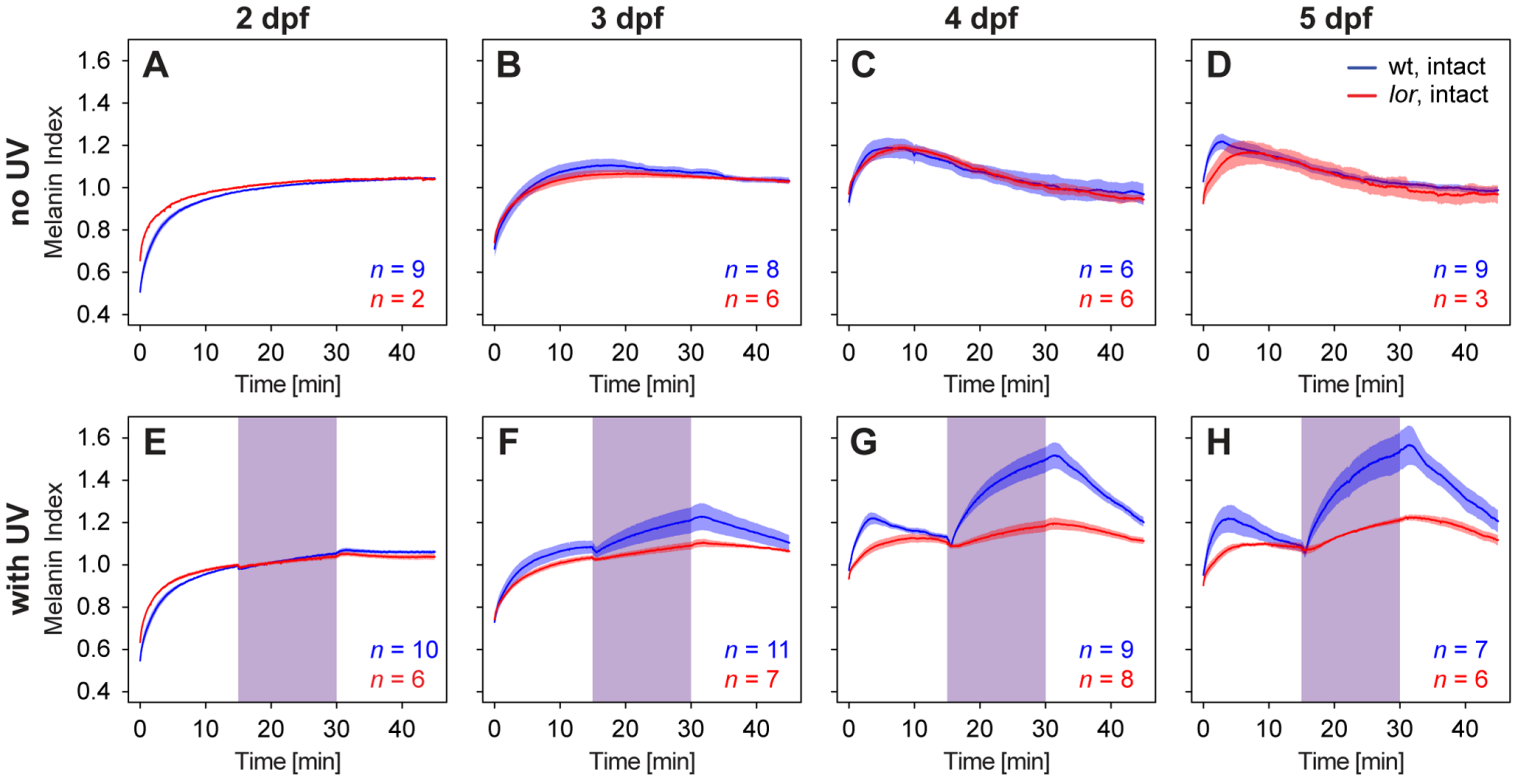
Influence of ocular short wavelength-sensitive (SWS) cone photoreceptors on pigmentation of larval zebrafish. lots-of-rods *(lor)* mutant zebrafish larvae largely lacking SWS cone photoreceptors show normal melanosome dispersion and aggregation under visible light exposure (A–D). However, the UV-light induced dispersion of melanosomes observed in wildtype fish of 3 dpf and older is strongly reduced (F–H). Values represent means ±1 SEM and are relative to the extent of pigmentation after 30 minutes light adaptation, i.e. a melanin index >1 means the larva is darker compared to the appearance after light adaptation, and vice versa, a value <1 means the larva appears paler compared to the appearance after light adaptation. Data of wildtype larvae is the same as shown in Fig. 2. For ease of comparison, all graphs are drawn at the same scale. Purple shaded areas mark the time interval when the UV light was on.

To test whether the UV sensitive cones in the larval retina are responsible for mediating the observed dispersal of melanosomes in response to UV illumination, we tested lots-of-rods *(lor)* mutants. In these larvae, a mutation in the transcription factor *tbx2b* leads to a near complete loss of UV-sensitive cones in favor of rods [21]. Whereas under normal light conditions *lor* mutants display the same pigmentation kinetics as wildtype fish (Fig. 3A–D), the dispersal of melanosomes in response to UV illumination is clearly reduced compared to wildtype larvae (Fig. 3F–H).

## Discussion

Zebrafish embryos at the age of 2 dpf do not show any visual behaviors [22], [23], and retinal opsins are not detectable until 51 hours post fertilization [24]. Given these facts, it is safe to conclude that embryonic eyes at 2 dpf are not yet functional. In combination with the results obtained from isolated tails, eyeless mutant larvae and blinded wildtype larvae, we suggest that the fast light-induced dispersion of melanosomes can solely be attributed to photoreceptors intrinsic to the melanophores. Lacking the ability to perceive the color of the local environment, it seems reasonable that the primary function of melanophores at early larval stages (i.e. in larvae younger than 3 dpf) is not to provide camouflage, but rather to protect the largely transparent animals from the harmful impact of potential UV irradiation. Due to our experimental setup, we were not able to measure the effect of visible light provided from above instead of from below, but we do not expect the angle of incidence being important at these larval stages.

In contrast, the photoreceptors in the retina of larvae at the age of 3 dpf and older are functional as shown by behavioral tests [22], [23], and the larvae are therefore not only able to perceive the color of their background, but owing to functional SWS cone photoreceptors are also able to experience the effective proportion of potentially harmful UV irradiation. At these larval stages, the melanophores seem to play a dual role: If no UV light is detected, the initial rapid dispersion of the melanosomes mediated by the melanophore intrinsic mechanism is counteracted by a slower, retina mediated aggregation, leading to a pale appearance on a bright background and hence providing camouflage. If, however, UV light is detected, the protective function from UV irradiation takes over, and melanosomes rapidly disperse, irrespective of the color of the local environment. In contrast to the situation found in younger larvae, we believe that the angle of incidence of visible light would matter in this situation: In order to provide useful visual background adaptation, the ratio between visible light hitting the eye directly from above (i.e. light detected in the ventral retina) and the proportion of light reflected by the background (i.e. light detected in the dorsal retina) should determine the magnitude of pigment dispersal.

Taken together, these results suggest that the primary function of melanosome dispersal has evolved as a protective adaption to prevent UV damage, which was only later co-opted for camouflage. The adaptive value of this reaction seems obvious, since zebrafish in the wild breed in shallow pools, where developing embryos are especially vulnerable to UV irradiation [25]. Strikingly, all *Danio* species exhibit identical pigmentation patterns at the age of 5 dpf, despite tremendous variations in adult pigmentation patterns [26]. Since all *Danio* species share similar habitats and therefore are subject to similar ecological constraints, this strongly suggests that the primary function of early larval pigmentation is the protection from UV-induced cell damage. It is also of note that the larval pigmentation patches nicely cover presumably particularly susceptible cell populations, i.e. the developing central nervous system and developing gonads. In addition, UV *and* visible light induced dispersion of pigments was also found in several species of echinoderms and crustaceans lacking visual background adaptation, adding evidence to our hypotheses that melanosome dispersion in response to light arose first as a means of UV protection and was only later co-opted for the purposes of camouflage [27]–[29].
